# *In vitro* Demonstration of Cancer Inhibiting Properties from Stratified Self-Organized Plasma-Liquid Interface

**DOI:** 10.1038/s41598-017-12454-9

**Published:** 2017-09-22

**Authors:** Zhitong Chen, Shiqiang Zhang, Igor Levchenko, Isak I. Beilis, Michael Keidar

**Affiliations:** 1 Department of Mechanical and Aerospace Engineering, The George Washington University, Washington, DC 20052 USA; 2 Plasma Sources and Applications Centre, National Institute of Education, Nanyang Technological University, 1 Nanyang Walks, Singapore, 637616 Singapore; 3 School of Chemistry Physics and Mechanical Engineering, Queensland University of Technology, Brisbane, QLD 4000 Australia; 4 School of Electrical Engineering, Tel Aviv University, Ramat Aviv, 69978 Israel

## Abstract

Experiments on plasma-liquid interaction and formation of thinly stratified self-organized patterns at plasma-liquid interface have revealed a nontrivial cancer-inhibiting capability of liquid media treated at self-organized interfacial patterns. A pronounced cancer suppressing activity towards at least two cancer cells, breast cancer MDA-MB-231 and human glioblastoma U87 cancer lines, was demonstrated *in vitro*. After a short treatment at the thinly stratified self-organized plasma-liquid interface pattern, the cancer inhibiting media demonstrate pronounced suppressing and apoptotic activities towards tumor cells. Importantly, this would have been impossible without interfacial stratification of plasma jet to thin (of several µm) current filaments, which plays a pivotal role in building up the cancer inhibition properties. Furthermore, thinly stratified, self-organized interfacial discharge is capable to efficiently control the ROS and RNS concentrations in the cancer-inhibiting media. In particular, abnormal ROS/RNS ratios are not achievable in discharges since they do not form stratified thin-filament patterns. Our findings could be tremendously important for understanding the cancer proliferation problem and hence, the potential of this approach in tackling the challenges of high cancer-induced mortality should be explored.

## Introduction

Despite tremendous efforts undertaken, cancer and cancer-related diseases still remain among the most dangerous and mortiferous abnormalities responsible for about 13% of human death cases, totally accounting for more than 7 million per year^1^. Cancer morbidity trends are now on the rise with 11 million deaths expected by 2030. Undoubtedly, cancer represents a problem of paramount importance and therefore sophisticated design approaches are vitally required to tackle this challenge. Different treatment methods including surgical techniques, medication drugs, and radiation-based approaches are routinely being used for cancer treatment. However, more progress is needed to cope with this stubborn problem and drastically drop down the mortality rate. Indeed, the problem still persists despite immense effort applied. Are there any ways to reverse the trend and find a really radical breakthrough? Apparently, novel approaches with unexpected, counterintuitive mechanisms should to be explored.

Plasma-based cancer therapy is one of the novel techniques which have recently demonstrated a significant potential in curing various types of cancers. It was revealed that cold and non-thermal atmospheric-pressure plasma indeed inhibits proliferation of human cancer cells^2,3^. Various mechanisms were proposed to explain this effect including the action of reactive oxygen and nitrogen species (ROS and RNS)^4,5^, and DNA damage^6^. In addition, it was shown that plasma action was selective towards the cancer cells while sparing normal cells, which is the main goal of anti-cancer therapies (“Holy Grail”). How do advance such a challenging problem? Unique processes at the self-organized interfaces could be a key to the production of novel, highly efficient tumor-inhibiting media. Moreover, self-organizational restructuration and patterning at the interfaces drastically widen the spectrum of the involved structural, physical and chemical processes, and thus opens new horizons to tackling tumors of various origins and locations.

Indeed, the idea was put forward recently to use a plasma-stimulated media^7–10^ to produce highly active complex material enriched with ROS, RNS and other active species to directly treat the tumor cells. Specific strategies amendable for adaptation of plasma-stimulated media were proposed but not yet realized^11^. Among them are the approaches based on miniaturization of discharge (and ultimately, involvement of micro-discharge and micro plasmas), and self-organizational phenomena. Self-organization is one of the key features of living matter. It is based on an entropy minimum principle and serves as a real engine for the development of animated nature. Moreover, it is also intrinsic to complex non-living systems; albeit not in such ubiquitous amount, self-organization still plays important role in some physical systems. An atmospheric pressure glow micro-discharge plasma with self-organized thinly stratified interface patterns (µAPGDP-SOTSIP) is a characteristic example of such self-organizing system^12,13^. The method was utilized in this work to prepare the cancer-killing plasma-activated therapeutic media.

In this work, we present a novel approach which demonstrates nontrivial cancer-inhibiting capabilities of spontaneous pattern-forming self-organization at the interface between atmospheric pressure glow discharge plasma and liquid media. After a short treatment at the self-organized plasma-liquid interface pattern, the cancer-inhibiting media has acquired a pronounced cancer-suppressing activity towards at least two kinds of human cancer cells, namely breast cancer MDA-MB-231 and human glioblastoma U87 cancer lines. Most important, the cancer-inhibiting media demonstrate tumor cell suppression and apoptotic activities, which would not otherwise be achievable without interfacial self-organization and the effect of bulk plasma jets without containing thin, µm-scaled current filaments. Apparently, the complex stratified self-organized interfacial patterns play a pivotal role in building up the cancer-inhibition properties. However, the specific mechanism between inhibition of the cancer proliferation and apoptosis is still not completely clear, and this paper aims mainly to

- *outline the major mechanism involved*,
- *disseminate this important finding, and*
- *attract attention of plasma-medical community to the urgent need to continue these studies*.

More efforts should be applied to fully discover the possible potential of this approach, tackling the challenge of high cancer-induced mortality and rising morbidity trends. A plausible mechanism is also discussed in terms of interaction of thin plasma filaments with gas and liquid causing accumulation of ROS/RNS and other species in unusual ratios and concentrations, forming potentially efficient anti-cancer cocktail.

## Results and Discussion

### Self-organization and plasma

Atmospheric pressure glow discharge and micro-discharge plasma with self-organized patterns is a growing research area, which attracts strong attentions of experts in various fields to deeply explore the physical mechanisms behind the self-organization^14,15^. Apart from the fascinating phenomena, the effect of µAPGDP-SOTSIP on tumor is among the most important feature of this system. Specifically, we have utilized here the µAPGDP-SOTSIP on a liquid surface (deionized (DI) water) to produce the plasma-activated therapeutic media under various regimes, and then apply them to MDA-MB-231and U87 cancer cells to conceptually reveal potential of this novel media in cancer therapy, and in particular, to demonstrate that the stratified interface pattern on a liquid surface plays a key role in the activation of therapeutic media. On the other hand, discharge mode transitions are shown to be leading to alteration of plasma-stimulated media and affect cancer cells. Plasma-activated therapeutic media has demonstrated more pronounced killing effect on U87 than MDA-MB-231 cancer cells. This is a largely unexpected results based on previous experience with these cell lines and their response to cold atmospheric plasma.

### Self-organized stratified patterns at plasma/liquid interface

The self-organized patterns and other phenomena are widely observed in both natural and anthropic fields, within diverse types of chemical, biological and physical systems including low temperature plasma^16,17^. µAPGDP-SOTSIP has gained an increasing attraction due to its unique features, ease ignition and maintenance, and diverse emerging applications^14^. Various plasma devices are able to produce self-organized plasma patterns, including DC micro-discharge devices with gas feed^18^, a pin water-anode glow discharge setups^19^, and dielectric barrier discharge-like devices^20^. Many key parameters such as gap length, excitation frequency and applied voltage were examined to study the effect on the pattern formation^21,22^. Moreover, numerical simulations were utilized to explain the basic physical mechanisms behind the formation of self-organized patterns^14,23^. We stress here that the µAPGDP-SOTSIP for the cancer cell treatment applications has not been reported yet. Although atmospheric pressure plasma studies for the biomedical applications and cancer therapy by reactive oxygen and nitrogen species (ROS and RNS)^24^ has been reported and demonstrated some potential.

### Results of self-organized plasma experiment

**Figure 1:**
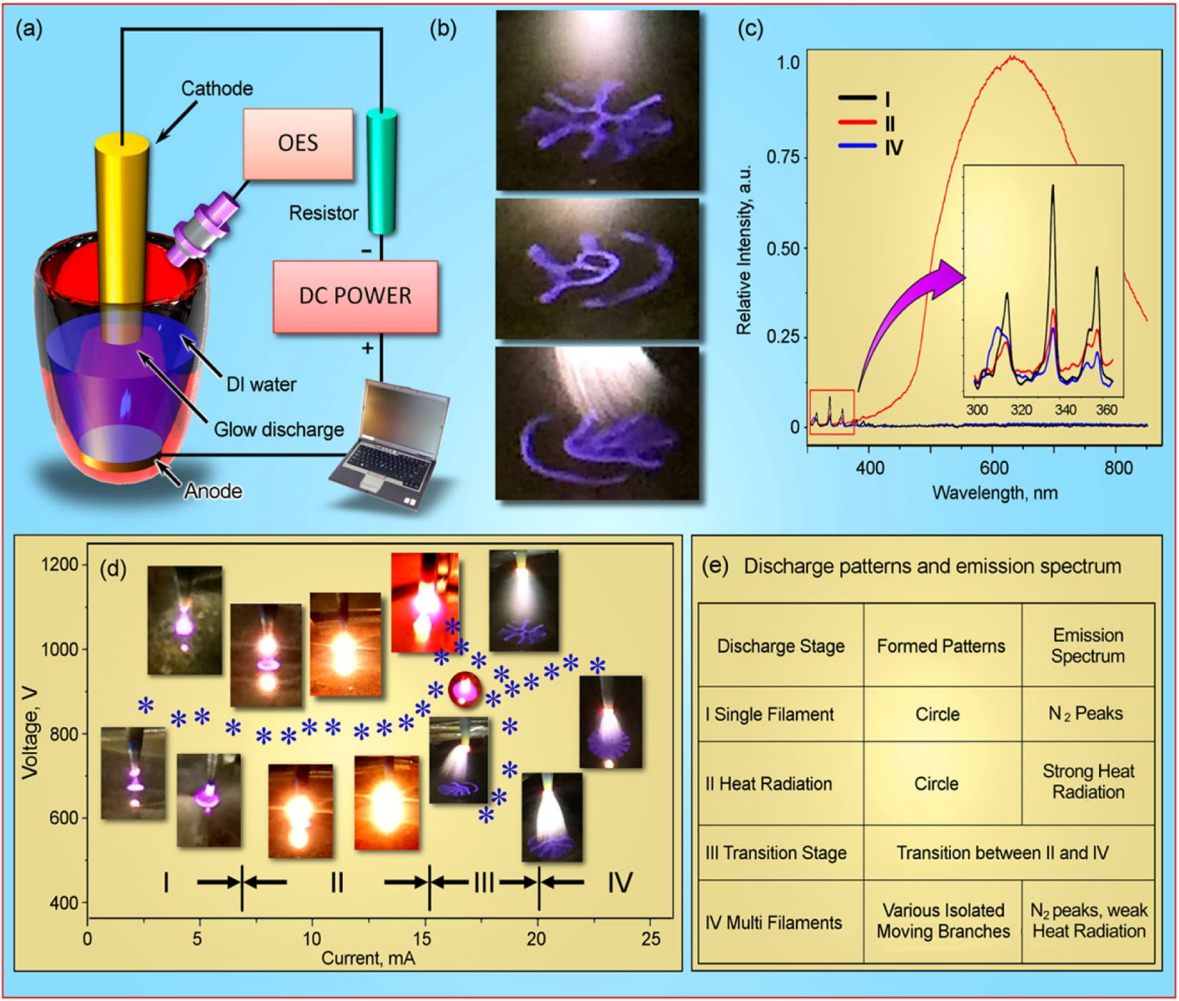
(**a**) Schematic representation of the atmospheric pressure glow micro-discharge setup. A small (several mm) gap between the cathode and surface of liquid accommodated a bunch of plasma. (**b**) Optical photographs of the discharge patterns above the therapeutic media during the activation process. The self-organized patterns have complex structure strongly depending on the voltage-current conditions. Stratification of plasma jet to a large number of thin current filaments is clearly visible at lower photograph of the panel; fine complex patters are also formed on the surface of liquid media; (**c**) Optical emission spectrum by the atmospheric pressure glow discharge above DI water taken using UV-visible-NIR in the 200–850 nm wavelength range. (**d**) Current-voltage dependence of the system with optical photographs of the self-organized stratified interface patterns. Four discharge stages could be clearly determined. (**e**) A table summarizing the relations between discharge stages, self-organized patterns and optical emission. Magnified optical photographs of the plasma patterns on surface, as well as photographs of the self-organized stratified interface patterns can be also found in the Supplementary Figures S3 and S4.

Figure 1b shows optical photographs of various self-organized stratified interface patterns with micro-discharges. Complex shapes consisting of radial and confocal lines of different density may be produced. Some elements of symmetry (both radial and axial) can be sometimes observed. The current-voltage characteristics and optical photographs discharge patterns above the DI water corresponding to the specific current/voltage conditions are shown in Fig. 1d. The whole current-voltage characteristic can be divided into four specific stages. At stage I (current not exceeding 7 mA) the discharge voltage and current are low, and the discharge pattern represent a single filament (i.e., contracted glow discharge) following the initial corona discharge. As the discharge voltages increases to a certain degree (stage II), the temperature of the tungsten cathode increases, resulting into drastically enhanced heat radiation, as clearly seen in Fig. 1c. At stage III, the discharge enters an unstable state where it alternates between two (II and IV) stages featuring strong heat radiation (stage II) and the multi-filament pattern (stage IV). Thus, it’s a chanllenge to investigate the effect of voltage on both cancer cells. A plausible reason for this behavior could be that the high temperature results in stronger thermionic rather than the secondary electron emission from a comparatively cold electrode. Thus the discharge flips from the multi-filament and heat radiation-supported stage where the thermionic emission is efficient enough to maintain the increased discharge current. At stage IV, the discharge stabilizes at the multi-filament stage and stretches out to large number of discharge filaments at liquid media surface. Various types of self-organized stratified interface patterns are formed above the liquid media surface, as shown in Fig. 1b and c. Magnified optical photographs of the microplasma patterns on surface, as well as photographs of the self-organized stratified interface patterns can be also found in the Supplementary Figures S3 and S4.

Figure 1c shows spectrum of the µAPGDP-SOTSIP at liquid media surface/plasma interface. Measurements were made for the three different stages, namely I, II, and IV (transient stage III is not characteristic due to an unstable current regime). The emission bands were identified by the technique described elsewhere^25^. Species at wavelengths 337, 358, and 381 nm were identified as a low-density N2 second-positive system (*C*3Π*u*−*B*3Π*g*). The intensity of emission at stage II is much higher than that of stages I and IV (see graphs at Fig. 1d) due to high discharge intensity at stage II. Based on the above considerations, we have selected stages (modes) I and IV as the basic platforms for the bio-oriented studies described below in detail.

### Liquid media activated by self-patterned plasma

We have studied the effect of µAPGDP-SOTSIP on the composition of liquid media at the two major discharge regimes, namely regime I (low-current mode when the discharge current does not exceed 7 mA), and mode IV which is characterized by well-shaped self-organized patterns at the plasma-liquid interface, as shown in Fig. 1c. Apparently, mode II (very bright radiation shown in Fig. 1c) might have very strong heat effect on the liquid medium being activated. This leads to a very large number of hardly predictable chemical reactions and eventually, unpredictable solution composition. On the other hand, mode III is unstable and does not represent a specific interest. In contrast, mode IV, featured very high current but potentially ‘soft’ effect due to the well pronounced self-organized patterns at the interfaces, represents a special interest to the media activation and further, to the effect on tumor cells.

Other types of organic or inorganic liquids can be applied in the device, such as saline solution, Ringer’s solution, Dulbecco modified eagle medium, etc. Self-organized plasma-activated DI water has the potential to be utilized as an oral medicine or to even be paired with other drugs or used as standalone drug. On the other hand, blood is a body fluid that delivers necessary substances, which is composed of blood cells suspended in blood plasma. Blood plasma constitutes 55% of blood fluid that is mostly water (92% by volume). It is very difficult to directly treat blood by plasma due to its coagulation and higher viscosity coefficient than that of the water. Thus, we can use strategy of stimulating DI water and injecting it into blood around tumor.

To avoid overheating, relative short treatment times of 12, 24, 36, 48, 60 seconds were chosen (further measurements have confirmed the adequacy of such a choice). Since these measurements are essentially concept-proof studies, the concentrations of the two main reactive species, H_2_O_2_ and NO_2_^−^, mostly important in bio- and medical applications (including cancer-killing therapy) were measured. We stress there that our study presents the first results of the effect of self-organized plasma with stratified interface patterns on RONS and human cancer cells. Thus, to clearly stress the unique features of the self-organized plasma, we have thoroughly compared the concentrations of H_2_O_2_ and NO_2_^−^ obtained in our experiments with the results described in literature for the different kinds of the discharges. The data is also listed in Table 1.

**Table 1:** H_2_O_2_ and NO_2_^−^ concentrations, as well as discharge conditions obtained in these experiments and compared similar results described in publications (experimental and simulations)^26–30^.

Ref.	H_2_O_2_, mmol/L	NO_2_^−^, mmol/L	Current/energy	Discharge type, working gas
This work	0.015	0.12–0.7	3…20 mA	DC with spatial pattern
ref.^29^	0.7	0.2	10–15 mA	
ref.^30^	2.8	0.23	300–400 kJ/L	Pulsed
	0.3	0.04	100–200 kJ/L	Corona
	0.1	0.04	300–400 kJ/L	Plasma jet
ref.^31^	9.8–1.2	0.2–0.5	10–18 A	DC transient spark discharge generated in atmospheric pressure
ref.^32^	0.2	0.1	20 A	Air
ref.^33^	8	2 × 10^−5^		Simulations

**Figure 2:**
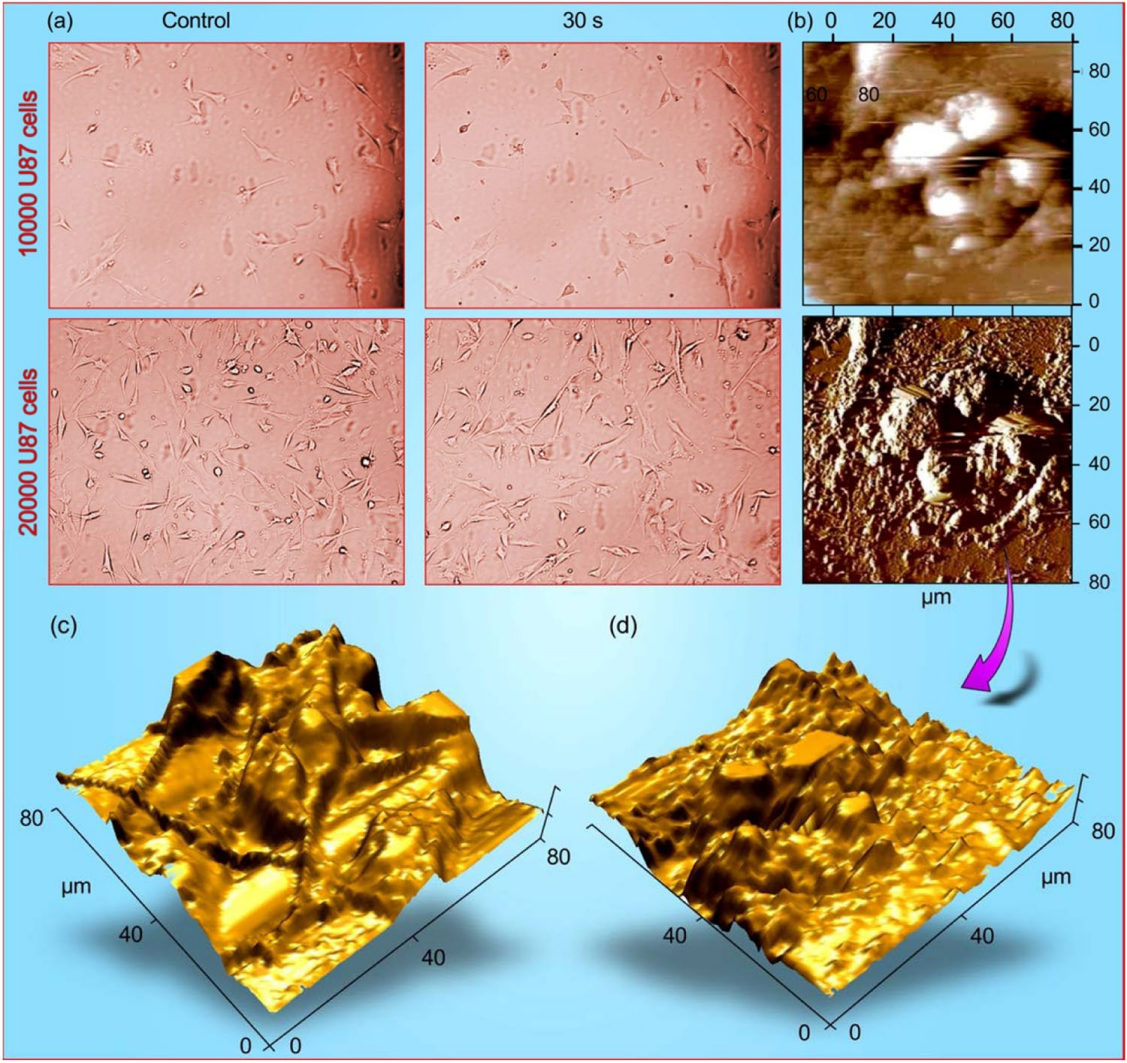
(**a**) Optical microscopy of 10,000 (upper row) and 20,000 (lower row) U87 cells, control and after 30 second treatment, after 24 h of incubation. (**b**) AFM images of U87 cells after 24 h of incubation and 30 s treatment. (**c**,**d**) Three-dimensional reconstruction of U87 cells before treatment (**c**) and after treatment (**d**), respectively (see more three-dimensional reconstruction of U87 cells in the Supplementary Figures S5 and S6).

**Figure 3:**
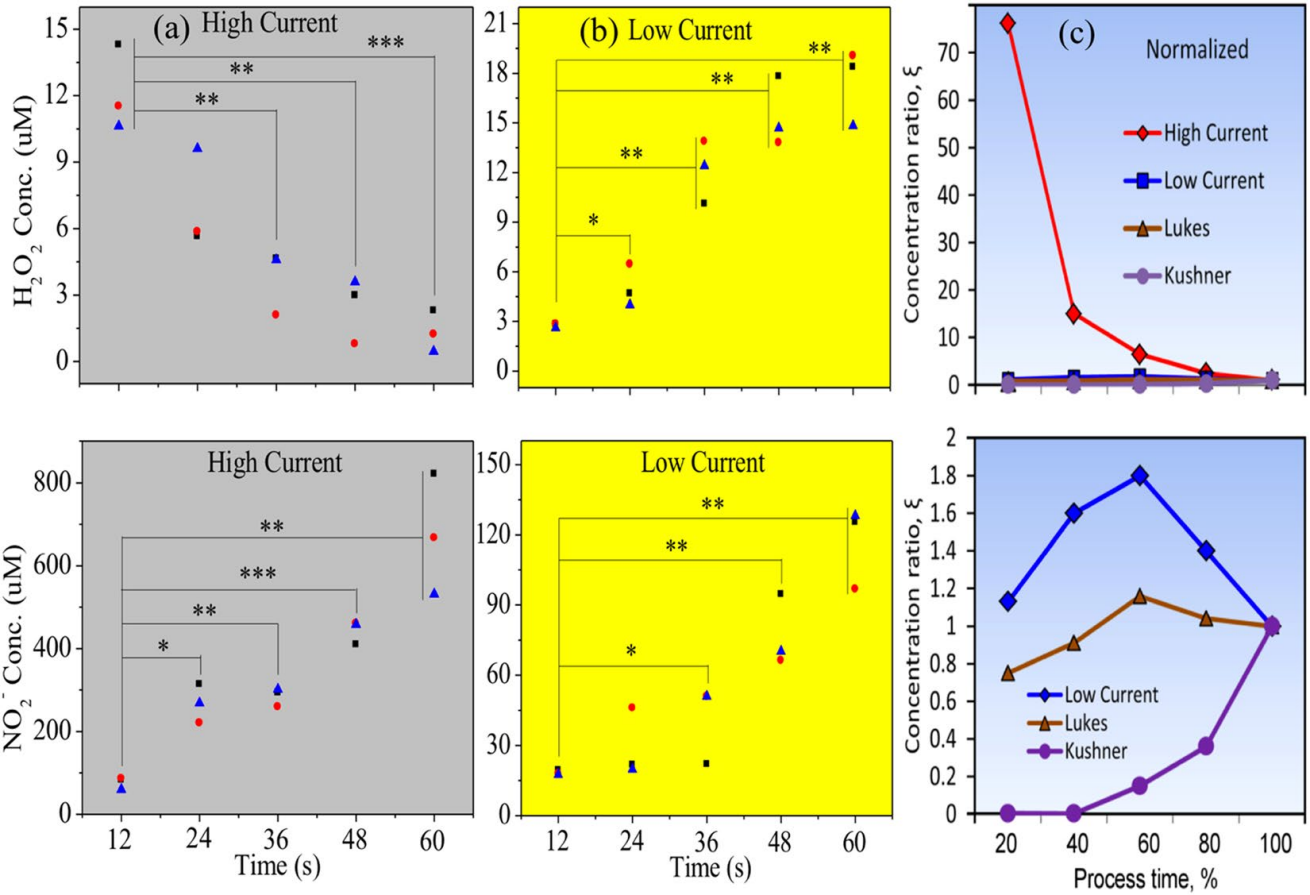
Time dependencies of ROS and RNS concentrations in liquid media treated with self-organized micro-discharge forming stratified interface patterns. (**a**) H_2_O_2_ concentration and NO_2_^−^ of high current (stage IV); (**b**) H_2_O_2_ concentration and NO_2_^−^ of low current (stage I). The concentration of H_2_O_2_ decreases and the concentration of NO_2_^−^ increases with the treatment time in high-current mode, while both H_2_O_2_ and NO_2_^−^ concentrations rise with the time. The absolute concentration of H_2_O_2_ features highest numbers at low-current mode, while the NO_2_^−^ concentration reaches strong maximum under the high-current mode. (**c**) Absolute (upper panel) and normalized (lower panel) H_2_O_2_/NO_2_^−^ ratios for the result obtained in this work, as well as in refs^31^ and ^32^, for comparison. Student’s t-test was performed, and the significance compared to the first bar is indicated as *p < 0.05, **p < 0.01, ***p < 0.001 (n = 3, (triangles, square and circles represent each time of experiments (total 3).

Figure 2 shows the typical optical and atomic force microscopy characterization results for the untreated and plasma-treated U87 cells. Decrease in number of cells with time, as well as destruction can be observed in these two types of characterization. The three-dimensional reconstructions of U87 cells (Fig. 2c and d, and Supplementary Figures S5 and S6) show the details of surface morphology of untreated and treated cells. The ROS and RNS contents in the µAPGDP-SOTSIP-treated liquid media (DI water) were determined. The time dependencies of the H_2_O_2_ and NO_2_^−^ concentrations in the µAPGDP-SOTSIP-stimulated liquid media produced by the two (low- and high-current) modes are shown in Fig. 3a and b. For the high-current mode (panel A), the concentration of H_2_O_2_ decreases while the concentration of NO_2_^−^ increases with the treatment time. The low-current mode (panel B) demonstrates opposite trend: both H_2_O_2_ and NO_2_^−^ concentrations rise with the time. Absolute concentrations also demonstrate different trends: while hydrogen peroxide H_2_O_2_ concentration features highest (17 μM versus 12 μM) numbers at low-current mode, the NO_2_^−^ concentration reaches strong maximum (650 μM versus 120 μM) under the high-current mode.

Examination of the processes taking place at the plasma-liquid interface allows suggesting the following mechanisms of H_2_O_2_, NO_2_^−^ and other RONS formation in plasma-stimulated media^31–33^:

1 $${{\rm{O}}}_{2}+{{\rm{e}}}^{-}\to {{\rm{O}}}_{2}^{-}$$

2 $${{\rm{O}}}_{2}^{-}+{{\rm{H}}}^{+}\to {{\rm{HO}}}_{{\rm{2}}}$$

3 $${{\rm{HO}}}_{{\rm{2}}}+{{\rm{e}}}^{-}\to {{\rm{HO}}}_{2}^{-}$$

4 $${{\rm{HO}}}_{2}^{-}+{{\rm{H}}}^{+}\to {{\rm{H}}}_{{\rm{2}}}{{\rm{O}}}_{2}$$

5 $${{\rm{H}}}_{{\rm{2}}}{\rm{O}}+{\rm{e}}\to {{\rm{H}}}_{{\rm{2}}}{{\rm{O}}}^{\ast }+{\rm{e}}$$

6 $${{\rm{H}}}_{{\rm{2}}}{{\rm{O}}}^{\ast }\to {}^{\ast }{\rm{O}}{\rm{H}}+{{\rm{H}}}^{\ast }$$

7 $${{\rm{H}}}^{\ast }+{{\rm{O}}}_{2}\to {}^{\ast }{\rm{O}}{\rm{H}}+{\rm{O}}$$

8 $${{\rm{O}}}_{2}+{\rm{e}}\to {\rm{O}}+{\rm{O}}+{\rm{e}}$$

9 $${\rm{O}}+{{\rm{H}}}_{2}{\rm{O}}\to 2{}^{\ast }{\rm{O}}{\rm{H}}$$

10 $${}^{\ast }{\rm{O}}{\rm{H}}+{\rm{O}}\to {}^{\ast }{\rm{H}}{\rm{O2}}$$

11 $${}^{\ast }{\rm{H}}{{\rm{O}}}_{2}\to {\rm{H}}++{{\rm{O}}}_{2}^{\ast -}$$

12 $${{\rm{O}}}_{2}^{\ast -}+{{\rm{O}}}_{2}^{\ast -}+2{{\rm{H}}}_{{\rm{2}}}{\rm{O}}\to {{\rm{H}}}_{{\rm{2}}}{{\rm{O}}}_{2}+{{\rm{O}}}_{2}+2{{\rm{OH}}}^{-}$$

13 $${{\rm{O}}}_{2}^{\ast -}+{{\rm{O}}}_{2}^{\ast -}+2{{\rm{H}}}^{+}\to {{\rm{H}}}_{2}{{\rm{O}}}_{2}+{{\rm{O}}}_{2}$$

14 $${\rm{O}}+{{\rm{O}}}_{2}\to {{\rm{O}}}_{3}$$

15 $${}_{\ast }{\rm{O}}{\rm{H}}+{}^{\ast }{\rm{O}}{\rm{H}}\to {{\rm{H}}}_{2}{{\rm{O}}}_{2}$$

16 $${{\rm{N}}}_{2}+{\rm{e}}\to 2{\rm{N}}+{\rm{e}}$$

17 $${\rm{O}}+{\rm{N}}\to {\rm{NO}}$$

18 $${\rm{NO}}+{\rm{O}}\to {{\rm{NO}}}_{2}$$

19 $$2{{\rm{NO}}}_{2}+{{\rm{H}}}_{{\rm{2}}}{\rm{O}}\to {{\rm{NO}}}_{2}^{-}+{{\rm{NO}}}_{3}^{-}+{{\rm{2H}}}^{+}$$

20 $$4{\rm{NO}}+2{{\rm{H}}}_{2}{\rm{O}}+{{\rm{O}}}_{2}\to 4{{\rm{H}}}^{+}+4{{\rm{NO}}}_{2}^{-}$$

Other possible reactions illustrating the routes of H_2_O_2_ and NO_2_^−^ formation in liquid and plasma are listed elsewhere^34^. According to these mechanisms, the concentration of H_2_O_2_ and NO_2_^−^ should increase with treatment time, while the concentration of H_2_O_2_ (at high current) was actually decreasing. The possible reason for this behavior is that the temperature of plasma at the high current mode is much higher than that at the low current conditions. Since hydrogen peroxide is thermodynamically unstable, its rate of decomposition increases with rising temperature^35^. Figure 3c shows the most unusual features of the ROS and RON measurements in liquid media stimulated by the self-organized plasma with stratified interface patterns. Whereas the normalized (upper panel) H_2_O_2_/NO_2_^−^ ratios for the result obtained in this work demonstrate strong drop with process time (i.e., the H_2_O_2_ concentration significantly exceeds that of NO_2_^−^), the results obtained for other kinds of plasmas (experimental studies by Machala *et al*.^26^ and theoretical findings by Takahashi *et al*.^27^, see also Table 1) demonstrate the numbers between zero and 1. The absolute graphs for the same ratio are shown in the lower panel. One can see that the ratios obtained in our experiments (up to 70) are very unusual in comparison with other kinds of plasma.

### Effect of plasma-activated media onto human cancer cells

**Figure 4:**
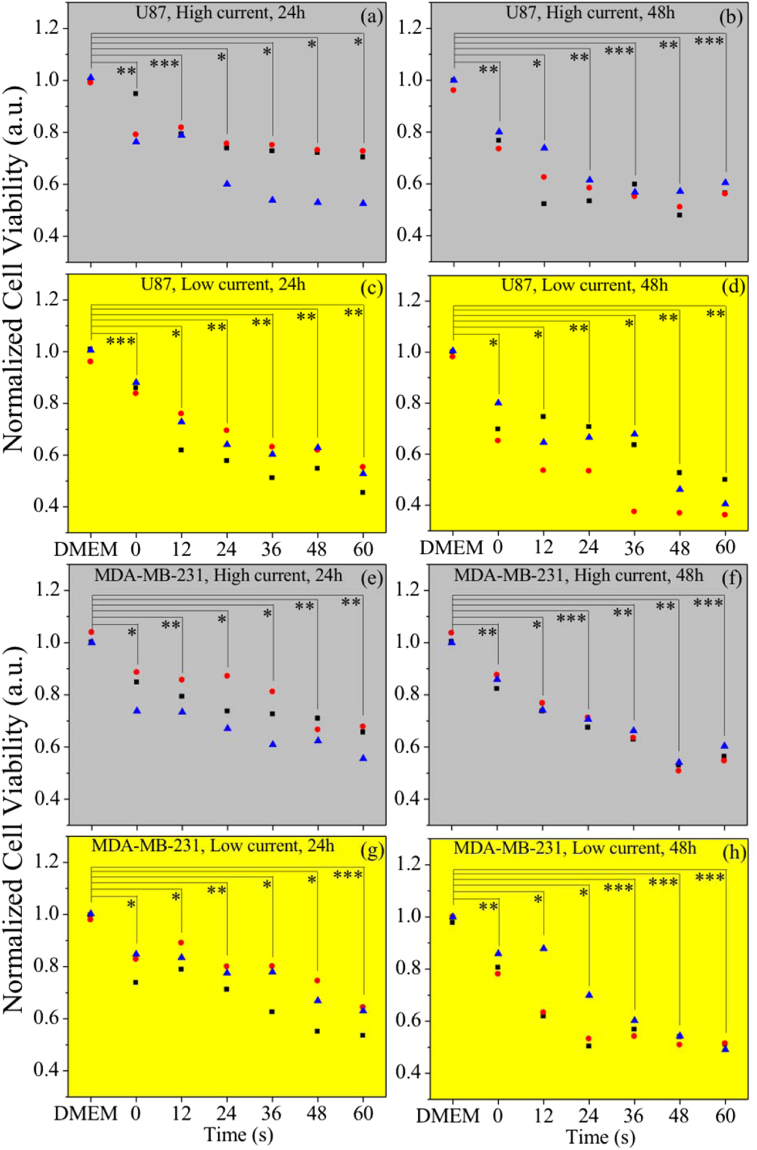
Effects of seven media: DMEM, DI water (0 second treatment), and five plasma-produced activated media (DI water activated by APGDP at high and low currents for 12, 24, 36, 48, and 60 seconds on viability of the U87 human glioblastoma cancer cells (**a**–**d**) and MDA-MB-231 human breast cancer cells (**e**–**h**) after 24 and 48 hours incubation. The percentages of surviving cells for each cell line were calculated relative to controls (DMEM = 1). Student’s t-test was performed, and the significance compared to the first bar is indicated as *p < 0.05, **p < 0.01, ***p < 0.001 (n = 3, (triangles, square and circles represent each time of experiments (total 3))).

To study the effect of the liquid media stimulated by the plasma with self-organized stratified interface pattern, two kinds of cancer cells were treated. The µAPGDP-SOTSIP-stimulated media was applied to the human glioblastoma (U87) and breast (MDA-MB-231) cancer cells, whereas the Dulbecco’s Modified Eagle’s Medium (DMEM) was used as the control platform. Cells were cultured in completed DMEM media, therefore, we added DMEM to cancer cells having no effect (control group). However, cells will be dead if they were cultured in DI water. On the other hand, we compared DI water with µAPGDP-SOTSIP activated DI water applied to cancer cells. µAPGDP-SOTSIP activated DI water had more effect on cancer cells than DI water, which means plasma solutions work from µAPGDP-SOTSIP not from DI water itself. Figure 4a–d show the viability of human glioblastoma cancer (U87) exposed to DMEM, deionized water treated by self-organized plasma at high (Fig. 4a and b) and low (Fig. 4c and d) currents for 0, 12, 24, 36, 48, and 60 seconds, incubated for 24 h (Fig. 4a and b) and for 48 h (Fig. 4c and d). The viability of the glioblastoma cancer cells incubated for 24 h in low current treated DI water was lower than the viability of cells exposed to incubated in media treated at high current. The viability of glioblastoma cancer cells incubated for 48 h in high current treated media decreased by approximately 23, 37, 42, 43, 48 and 42%, respectively (average). For the case of low current treated media, the viability of cells decreased by approximately 28, 36, 37, 44, 55, and 58%, respectively (average). The minimum viability of U87 cells was detected for the media treated by high current (mode IV - self-organization at the liquid-plasma interface) incubated for 48 seconds. In all the U87 cell line experiments, cell viability decreased with the increasing of the plasma treatment time. Due to MTT curves flattening after 24 s of voltage application, the statistical analysis have been performed for U87 cancer cells between high and low current models for 24 h (36 s (*P < 0.05), 48 s (*P < 0.05), and 60 s (P > 0.05)) and for 48 h (36 s (*P < 0.05), 48 s (*P < 0.05), and 60 s (**P < 0.01)).

Figure 4e–h show the viability of the human breast cancer cells (MDA-MB-231) exposed to DMEM, deionized water, and plasma-activated media after incubation for 24 and 48 h. The viability of breast cancer cells incubated in plasma media activated at low current (i.e., in mode I without self-organize patterns) is in general lower than that of cells incubated in high current plasma-activated media (in the self-organized pattern mode). The viability of breast cancer cells incubated for 48 h in media activated at low current always decreases with treatment duration, while the viability of cells incubated in media activated at high current initially decreases, and then increases slightly. Due to MTT curves flattening after 24 s of voltage application, the statistical analysis have been performed for MDA-MB-231 cancer cells between high and low current models for 24 h (36 s (P > 0.05), 48 s (*P < 0.05), and 60 s (***P < 0.001)) and for 48 h (36 s (**P < 0.01), 48 s (P > 0.05), and 60 s (*P < 0.05)).

The comparison of the effect of plasma-stimulated media on human breast and glioblastoma cancer cells reveals a more pronounced plasma effect on the viability of glioblastoma cancer cells. It’s known that ROS can induce apoptosis and necrosis, whereas RNS induces damage to DNA resulting into cell death^36,37^. Hence, the trend of U87 and MDA-MB-231 cancer cells death after incubation on the media activated by self-organized plasma at low current can be attributed to the increase of ROS and RNS concentrations with treatment time. Moreover, a synergistic effect of RNS and ROS could play an important role in the apoptosis effect^38^. When the therapeutic media is processed at high current (i.e., self-organized plasma patterns are established at the plasma-liquid interface), the viability trend for both types of cells might depend on ROS more than RNS because the highest concentration of RNS did not result into lowest cell viability. Comparing the viability behavior for both cell types incubated in the high- and low current plasma-activated media, we can indicate that the elevated ROS concentration may play more important role than the RNS induced apoptosis. Besides, we should point out that lower viability of U87 cells versus that of breast cancer cells is an unexpected and very important finding (given that the U87 cells are considered to be more resilient), and thus, more detailed studies should be encouraged.

While we have tested only two types of cancer cells in this paper, the self-organized plasma-activated media should we believe work for more types of cancer cells. The proposed platform and the technique based on the application of self-organized, stratified plasmas for activation of anti-cancer properties will have a significant potential, and apparently, test on the other types of cancer cells will be the next stage of exploring this approach. On the other hand, the results obtained for quite different types of cells, breast cancer MDA-MB-231 and human glioblastoma U87 cancer lines, clearly indicate the outstanding potential of this technique.

Apparently, other types of organic or inorganic liquids such as saline solution, Ringer’s solution, Dulbecco modified eagle medium, etc. can be potentially utilized in this technique. Since just water (after plasma treatment) play a key role in the anti-cancer activity, other liquids including many biologically compatible ones could be expected to have similar behavior, and this even widens the applicability of our technique.

It should be noted that while plasma contains a large variety of reactive oxygen species (ROS), reactive nitrogen species (RNS), charged particles, electrons etc.^39^, the main stress here wa done to the H_2_O_2_ and NO_2_- spcies. It is well known that ROS and RNS play extremely important roles in cancer therapy due to their ability to induce apoptosis and damage proteins, lipids, and DNA. Currently, scientists determine H_2_O_2_ and NO_2_- as the key long life species with the anti-cancer capabilities. Apparently, it was a quite logical solution to first try just these species in their anti-cancer properties in the proposed technique. Furthermore, systematic studies on the role of other species would possible *open even more capabilities of the method*, but it apparently require a consolidated effort of many experts.

In summary, this paper presents the results of new studies revealing the role and significant potential of self-organization at the interface between self-organization plasma and liquid, capable of efficiently inhibiting the growth and proliferation of at least two kinds of human cancer cells, namely breast cancer MDA-MB-231 and human glioblastoma U87 cancer lines. Based on the current-voltage characteristics and optical self-organized patterns at the liquid surface, we have defined the four quite different micro-discharge modes and demonstrated that the activation under self-organized conditions plays a pivotal role in the synthesis of novel cancer-active media. Thus, we have discovered a novel, unexplored mechanism featuring pronounced capabilities for tumor inhibition, which could have a great anti-cancer potential but still is not well understood. Moreover, we have demonstrated that the self-organized micro-discharge is capable of efficient controlling the ROS and RNS concentrations in the therapeutically media, and in particular, the ROS/RNS ratios not achievable by other types of discharges could be obtained. Our findings could be extremely important for handling the cancer proliferation problem. Therefore, it should be brought to light to attract attentions of the researchers to explore the possible potential of this approach, tackling the challenge of high cancer-induced mortality and rising morbidity trends.

## Material and Methods

### Novel self-patterned glow micro-discharge plasma device

Figure 1a shows a schematic representation of the glow atmospheric pressure micro-discharge setup capable of producing well-defined self-organized stratified interface patterns at the liquid media surface/plasma interface. The media-producing discharge and self-organized interfacial patterning were organized as follows. Anode (thin copper plate, thickness d = 0.2 mm, Ø = 22 mm) was placed at the bottom of a glass-made treat well. Above the plate, 6 ml of deionized water was added to the well. The tungsten cathode of Ø = 2 mm was then installed above the water surface. A ballast resistor (90 kΩ) was connected between the cathode and the direct current (DC) power supply unit (Power Design, Model 1570 A, 1–3012 V, 40 mA). Voltage is applied between cathode and liquid-immersed anode, and a small (1–2 mm) gap between the cathode and surface of liquid accommodated a bunch of plasma. DI water was treated by self-patterned glow discharge plasma with 12, 24, 36, 48, and 60 seconds to obtain plasma solutions applied to cancer cells.

Photographs of the experimental setup, as well as stratified microplasma jet interacting with the liquid surface in the form of thin current filaments creating surficial pattern can be seen in Supplementary Figures S1 and S2. Besides, Supplementary video related to this article showing the discharge and formation of self-organized pattern can be found at the Publisher’s website.

### Optical emission spectra measurement

UV-visible-NIR, a range of wavelength 200–850 nm, was investigated on plasma to detect various RNS and ROS (nitrogen [N_2_], nitric oxide [–NO], nitrogen cation [N^+2^], atomic oxygen [O], and hydroxyl radical [–OH]). The spectrometer and the detection probe were purchased from Stellar Net Inc. In order to measure the radius of the plasma in DI water, a transparent glass plate was used to replace part of container. The optical probe was placed at a distance of 2 cm in front of plasma jet nozzle. Integration time of the collecting data was set to 100 ms.

### Cell culture

The human breast cancer cell line (MDA-MB-231) and glioblastoma cancer cell line (U87) were provided by Dr. Zhang’s lab and Dr. Young’s lab at the George Washington University, respectively. Both cells were cultured in Dulbecco’s Modified Eagle Medium (DMEM, Life Technologies) supplemented with 10% (v/v) fetal bovine serum (Atlantic Biologicals) and 1% (v/v) penicillin and streptomycin (Life Technologies). Cultures were maintained at 37 °C in a humidified incubator containing 5% (v/v) CO_2_. Cells were in non-linear growth at baseline, the data could be fitted to the exponential growth curve.

### Evaluation of ROS concentration

Fluorimetric Hydrogen Peroxide Assay Kit (Sigma-Aldrich) was used for measuring the amount of H_2_O_2_. A detailed protocol can be found on the Sigma-Aldrich website. Briefly, we added 50 *μ*l of standard curves samples, controls, and experimental samples (DI water treated by self-patterned glow discharge plasma with 12, 24, 36, 48, and 60 seconds) to the 96-well flat-bottom black plates, and then added 50 *μ*l of Master Mix to each of wells. We incubated the plates for 20 min at room temperature protected from light on and measured fluorescence by Synergy H1 Hybrid Multi-Mode Microplate Reader at Ex/Em: 540/590 nm.

### Evaluation of RNS concentration

RNS level were determined by using the Griess Reagent System (Promega Corporation) according to the instructions provided by the manufacturer. Briefly, we added 50 *μ*l of standard curves samples, controls, and experimental samples to the 96-well flat-bottom plates. Then dispense 50 *μ*l of the Sulfanilamide Solution to all samples and incubate 5–10 minutes at room temperature. Finally, dispense 50 *μ*l of the NED solution to all wells and incubate at room temperature 5–10 minutes. The absorbance was measured at 540 nm by Synergy H1 Hybrid Multi-Mode Microplate Reader.

### Characterization cell viability of MDA-MB-231 and U87

The cells were plated in 96-well flat-bottom microplates at a density of 3000 cells per well in 100 *μ*L of complete culture medium. Cells were incubated for 24 hours to ensure proper cell adherence and stability. On day 2, 40 *μ*L of and experimental samples (DI water treated by self-patterned glow discharge plasma with 12, 24, 36, 48, and 60 seconds) were added to cells, and 40 *μ*l of DMEM and DI water was added to the cells. Cells were further incubated at 37 °C for 24 and 48 hours. After 24 and 48 hours’ incubation, all cells media were discarded and then 100 μL of MTT solution (3-(4, 5-dimethylthiazol-2-yl)-2,5- diphenyltetrazolium bromide (Sigma- Aldrich) mixed with DMEM (5 mg/10 mL)) was added to each well followed by a 3-hour incubation. The MTT solution was discarded and 100 μL of solvent ((0.4% (v/v) hydrogen chloride in anhydrous isopropanol) was added to the wells. The absorbance of the purple solution was recorded at 570 nm with the Synergy H1 Hybrid Multi-Mode Microplate Reader. For 24 hours’ cell viability, control group is higher than other groups, which means cells in other groups die depending on dose. Compared 24 with 48 hours, cells in control group at 48 hours is much higher than 24 hours, which means cells in control group always growing. In other groups, cells dying depends on dose.

No linearity of the MTT assay was observed. Before measuring cell metabolic activity, cell cultured media would be replaced by MTT assay (mixed with DMEM). Cells with MTT solution were incubated for just three hours, then replaced by solvent ((0.4% (v/v) HCl in anhydrous isopropanol).

We should mention here that the MTT is a standard assay technique widely used in plasma medicine community^40,41]^. The MTT technique applied in this work has demonstrated a clear trend, thus definitely supporting our major findings that we believe should be demonstrated to the community due to their importance.

### Statistical analysis

All results were presented as mean ± standard deviation plotted using Origin 8. Student’s t-test was applied to check the statistical significance (*p < 0.05, **p < 0.01, ***p < 0.001).

## Electronic supplementary material

Supplementary information
