# Supplementary material for: In vitro Demonstration of Cancer Inhibiting Properties from Stratified Self-Organized Plasma-Liquid Interface
Source: Sci Rep. 2017 Sep 22;7:12163. doi: 10.1038/s41598-017-12454-9 (PMC5610191; doi:10.1038/s41598-017-12454-9)
Supplement: Supplementary file 1 — Supplementary information [file 41598_2017_12454_MOESM1_ESM.doc]

**Supplementary Information**

*In vitro* Demonstration of Cancer Inhibiting Properties from Stratified Self-Organized Micro-Discharge Plasma-Liquid Interface

*Zhitong Chen^1^, Shiqiang Zhang^1^, Igor Levchenko^2,3^, Isak I. Beilis^4^, Michael Keidar1,[[1]](#footnote-2)**

^1^ Department of Mechanical and Aerospace Engineering, The George Washington University, Washington, DC 20052, USA

^2^ Plasma Sources and Applications Centre, National Institute of Education, Nanyang Technological University, 1 Nanyang Walks, 637616, Singapore

^3^ School of Chemistry Physics and Mechanical Engineering, Queensland University of Technology, Brisbane QLD 4000, Australia

^4^ [School of Electrical Engineering](http://www.eng.tau.ac.il/index.php?option=com_content&view=article&id=170&Itemid=192&language=en-GB), [Tel Aviv University](http://www.tau.ac.il/), Ramat Aviv 69978, Israel

**TABLE OF CONTENTS**

1. **Fig. S1:** Photograph of the experimental setup

2. **Fig. S2:** Two different regimes of plasma interaction with liquid media

3. **Fig. S3:** Optical photographs of the discharge patterns above the therapeutic media during the activation process

4. **Fig. S4:** Magnified optical views of the patterns on liquid media – plasma interface

5. **Fig. S5.** Three-dimensional reconstructions of U87 cells after treatment for 24 h.

6. **Fig. S6:** Three-dimensional reconstructions of U87 cells after treatment for 47 and 48 h


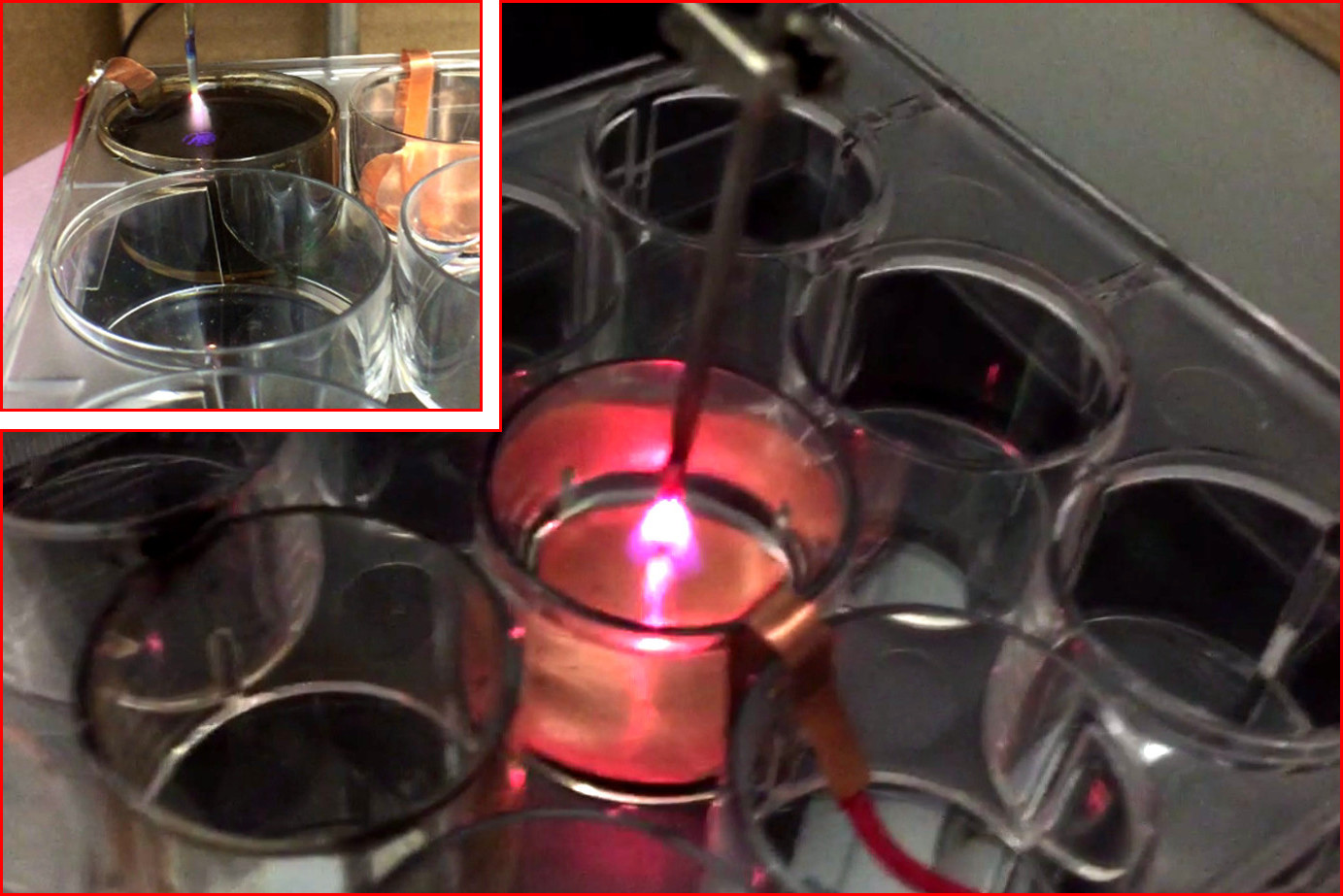


**Fig. S1.** Photographs of the experimental setup.

**Left upper panel**: General view of the electrode and discharge over the liquid media.
**Right panel**: Atmospheric plasma interacts with the surface of liquid media.


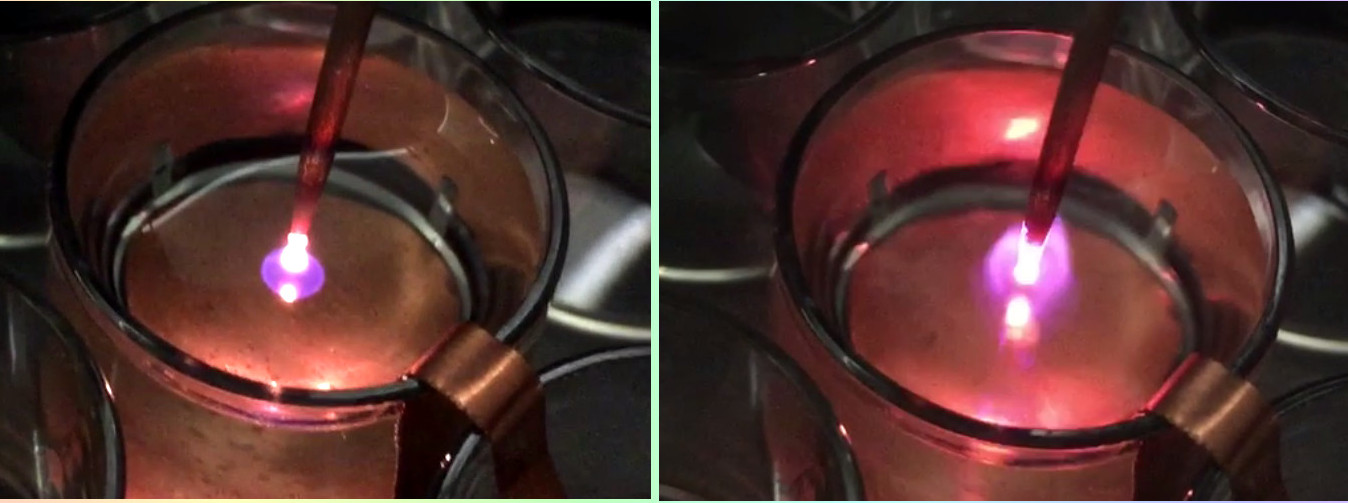


**Fig. S2.** Two different regimes of plasma interaction with liquid media:
Left panel, steady regime with pattern established at the plasma-liquid interface;
Right panel, transition regime without pattern.


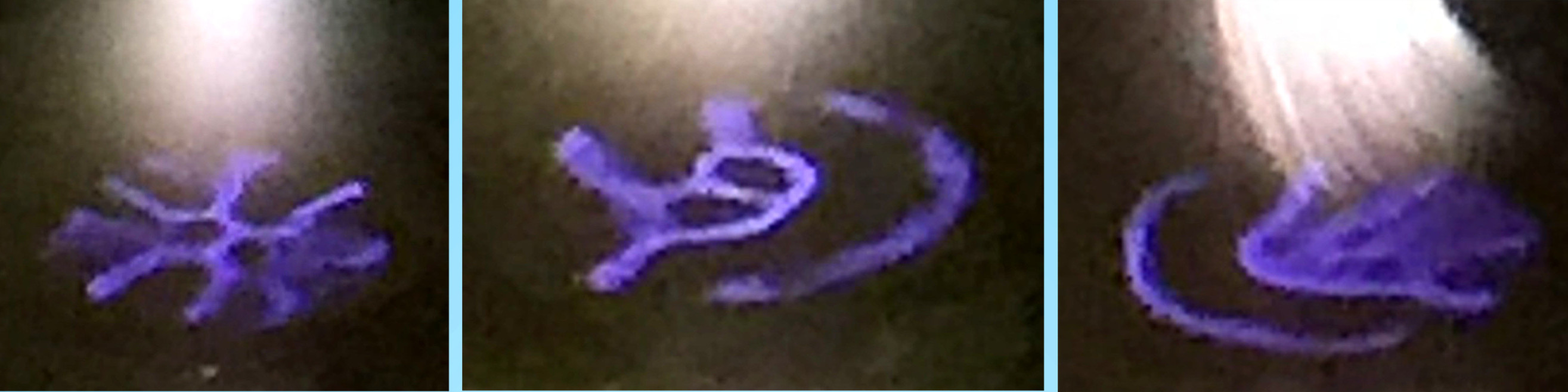


**Fig. S3.** Magnified optical photographs of the discharge patterns above the therapeutic media during the activation process. The self-organized patterns have complex structure strongly depending on the voltage-current conditions.


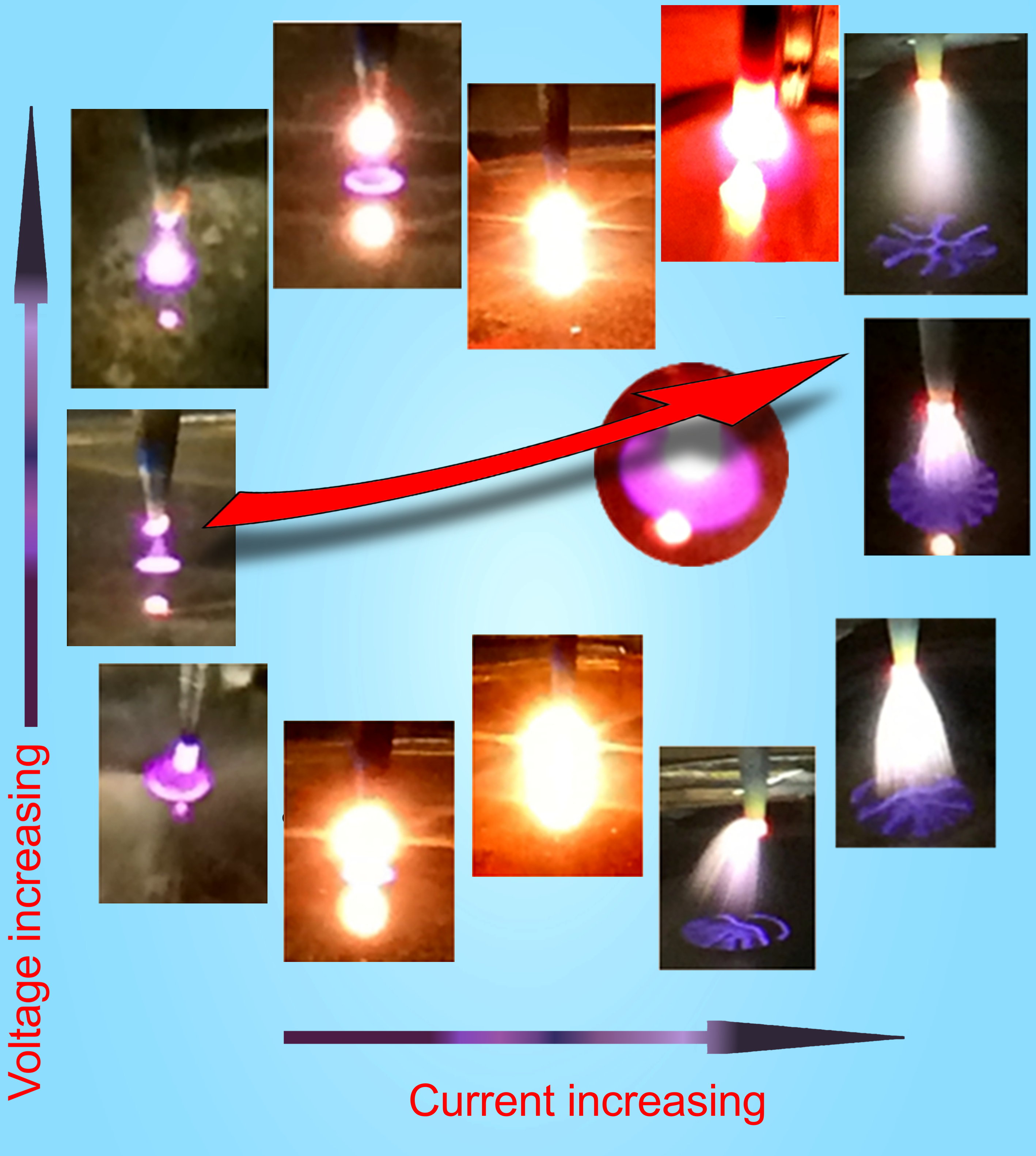


**Fig. S4.** Magnified optical views of the patterns on liquid media – plasma interface.


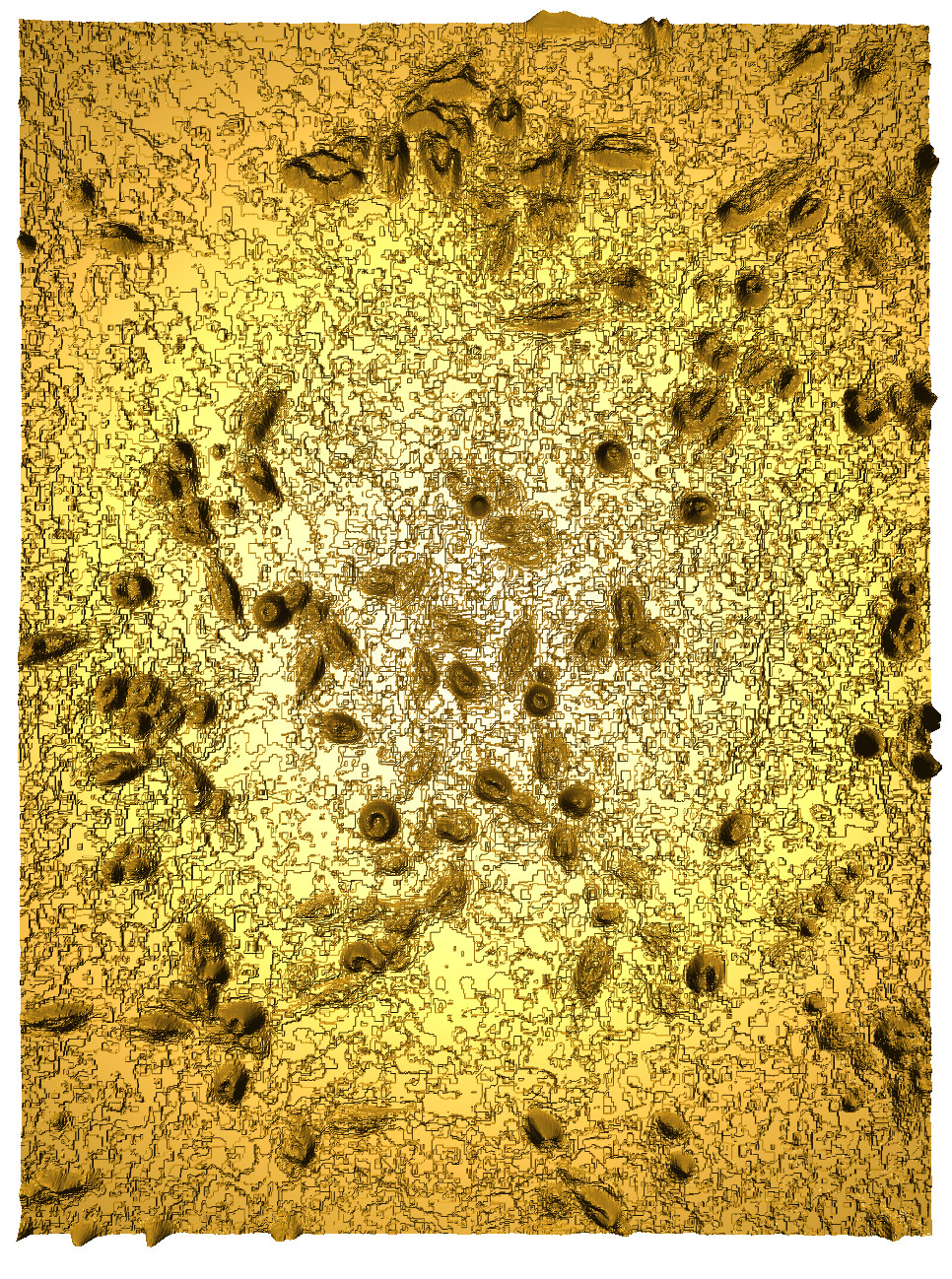


**Fig. S5.** Three-dimensional reconstructions of U87 cells after treatment for 24 h.

Sample size is 100 µm, feature heights are conditional.


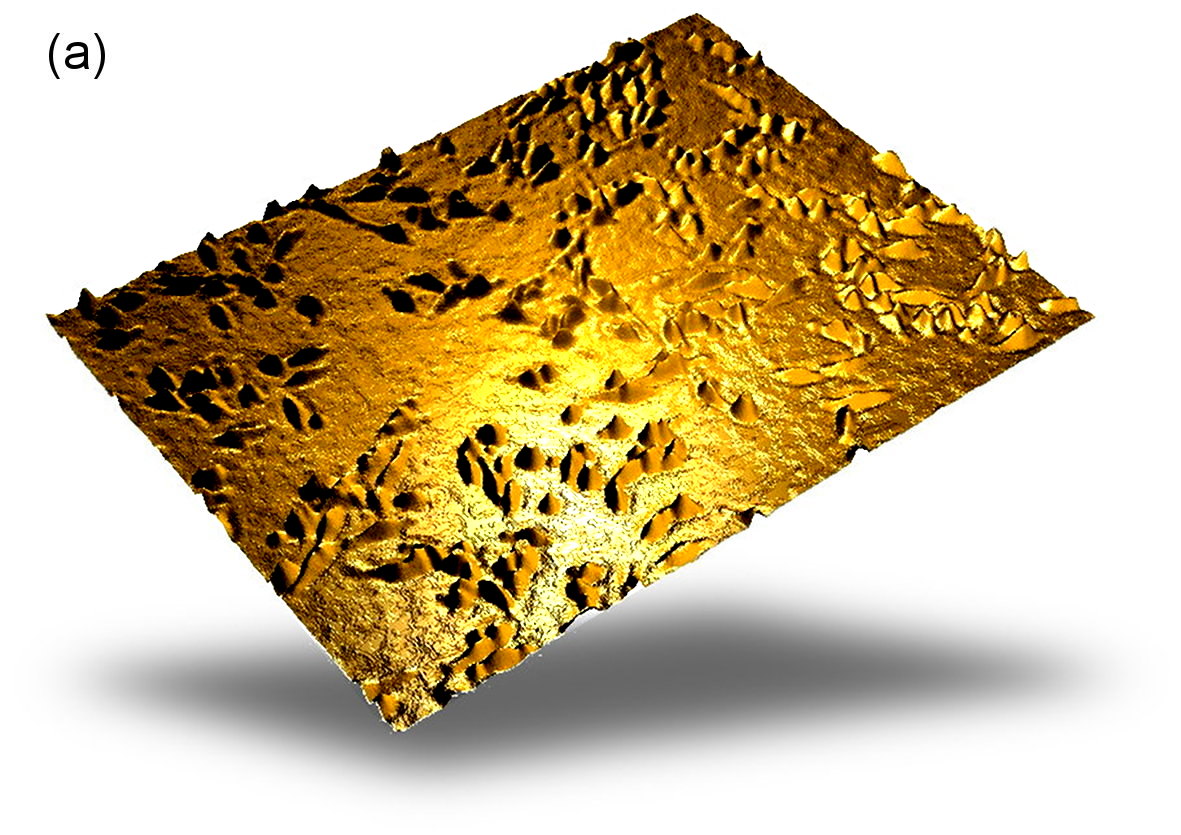


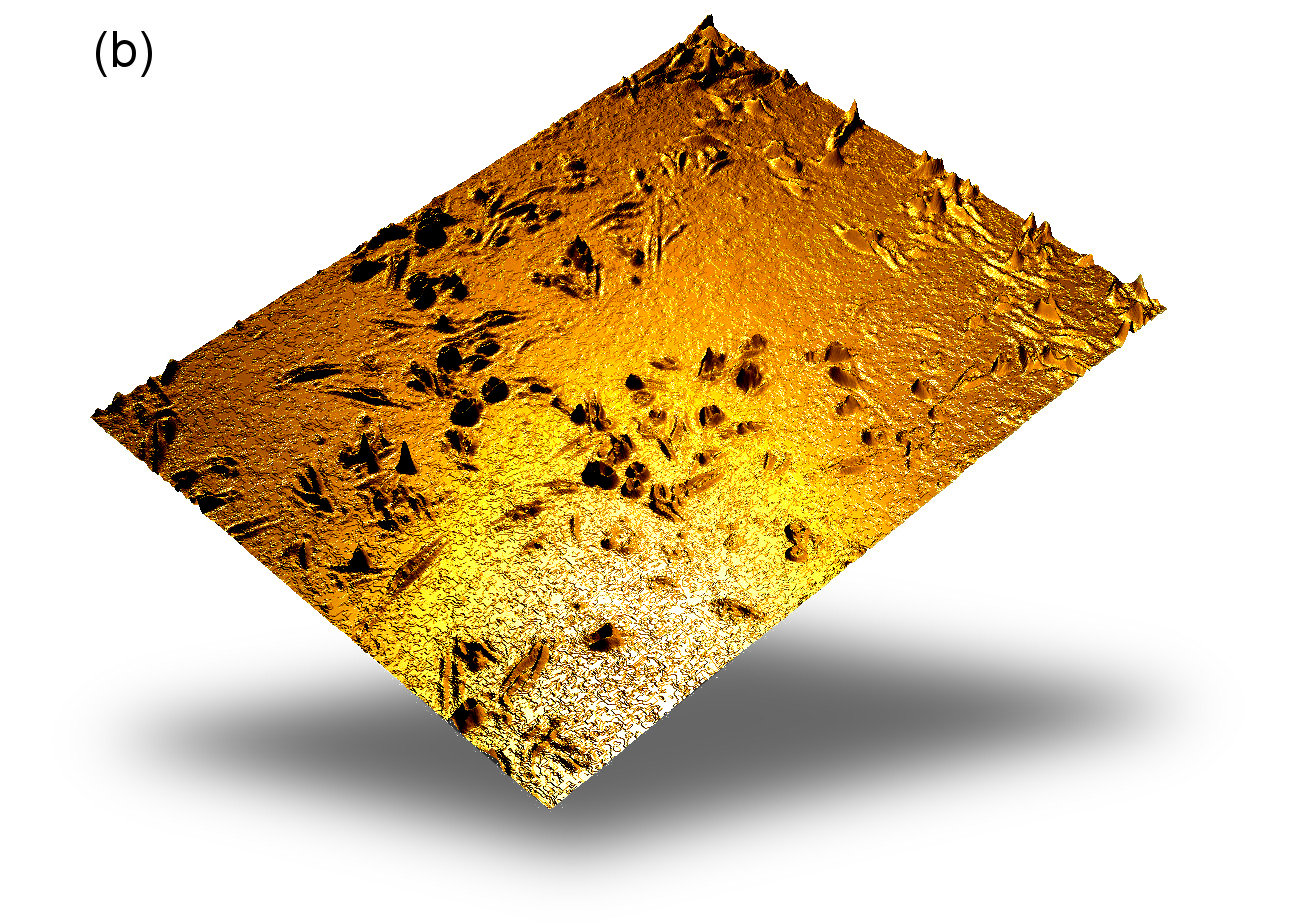


**Fig. S6.** Three-dimensional reconstructions of U87 cells after treatment for (a) 47 h and (b) 48 h, respectively. Sample size is 100 µm, feature heights are conditional.

1. ^*^ Corresponding Author:

   E–mail address: [zhitongchen@gwu.edu](mailto:zhitongchen@gwu.edu) (Z. Chen), Igor.Levchenko@qut.edu.au (I. Levchenko), [beilis@eng.tau.ac.il](mailto:beilis@eng.tau.ac.il) (I. Beilis), [keidar@gwu.edu](mailto:keidar@gwu.edu) (M. Keidar) [↑](#footnote-ref-2)
